# Ophthalmologic alterations in cutis marmorata telangiectatica congenita: a series of cases

**DOI:** 10.5935/0004-2749.20200064

**Published:** 2020

**Authors:** Lana Sayuri Makita, Bernardo Carvalho Muniz, Flávio Mac Cord Medina

**Affiliations:** 1 Departamento de Oftalmologia, Universidade do Estado do Rio de Janeiro, Rio de Janeiro, RJ, Brasil; 2 Departamento de Radiologia, Instituto Estadual do Cérebro Paulo Niemeyer, Rio de Janeiro, RJ, Brasil

**Keywords:** Skin diseases, vascular, Telangiectasias/congenital, Eye manifestations, Dermatopatias vasculares, Telangiectasia/congênito, Manifestações oculares

## Abstract

Cutis marmorata telangiectasia congenita is a rare cutaneous vascular disorder that may be associated with different systemic manifestations like body asymmetry, cutaneous, ophthalmologic, vascular, and neurological manifestations. We describe ophthalmologic alterations found in three patients with cutis marmorata telangiectatica congenita highlighting the rare retinal manifestations.

## Abstract (pt)

A Cutis marmorata telangiectasica congênita é uma desordem vascular cutânea rara que pode estar associada a diferentes manifestações sistêmicas, como assimetria corporal, alterações cutâneas, oftalmológicas, vasculares e neurológicas. Descrevemos alterações oftalmológicas encontradas em três pacientes com cutis marmorata telangiectasia congênita, destacando as raras manifestações retinianas.

## INTRODUCTION

Cutis marmorata telangiectatica congenita (CMTC) is a rare sporadic cutaneous vascular disorder, described in 1922 by Van Lohuizen, usually present at birth and characterized by persistent cutaneous reticulate erythema. CMTC can be associated with telangiectasias, phlebectasias (venous dilations), ulcerations, cutaneous atrophies, nevus flammeus, and others^(1-4)^. Ophthalmologic involvement is rare, with congenital glaucoma being the most frequent, but can also cause retinal vascular alterations, cataract, or retinoblastoma^(1,5)^. The pathogenesis of the disease is unknown, and the prognosis is good in cases without secondary complications^(3)^. In this series of cases, we present three patients with CMTC and ophthalmological alterations.

## CASE 1

**Figure 1:**
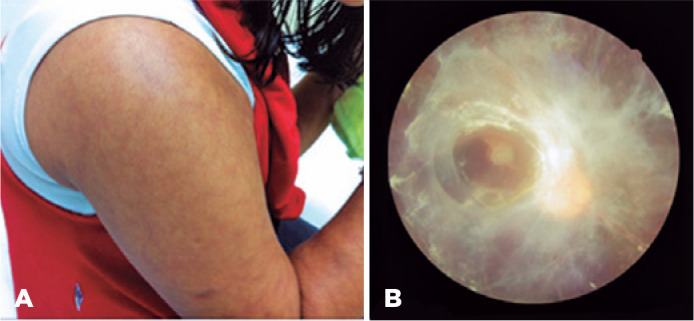
A) Livedo reticularis. B) Retinal examination of right eye revealing extensive fibrovascular membrane with retinal traction in vascular arches.

A 17-year-old female student with CMTC and visual loss in the left eye (OS) since childhood, started presenting progressive visual loss in the right eye (OD) one year before coming to see us. She lacked histories of surgery, ocular trauma, associated comorbidities, drug use, or similar cases in her family. Upon examination, we found bilateral and symmetrical congenital livedo reticularis in her trunk and limbs, with a better-corrected visual acuity at 20/60 in OD and no light perception in OS. We found no biomicroscopy alterations in the OD, and phthisis bulbi with white cataract and posterior synechiae in the OS. The intraocular pressures (IOPs) were 26 mmHg in the OD and 15 mmHg in the OS. Fundoscopic OD images showed an extensive fibrovascular membrane with retinal traction in vascular arches and macular atrophy (Figure 1); the OS fundi were undetectable due to cataract. We scheduled her for follow-ups to evaluate the retinal traction progression.

## CASE 2

**Figure 2:**
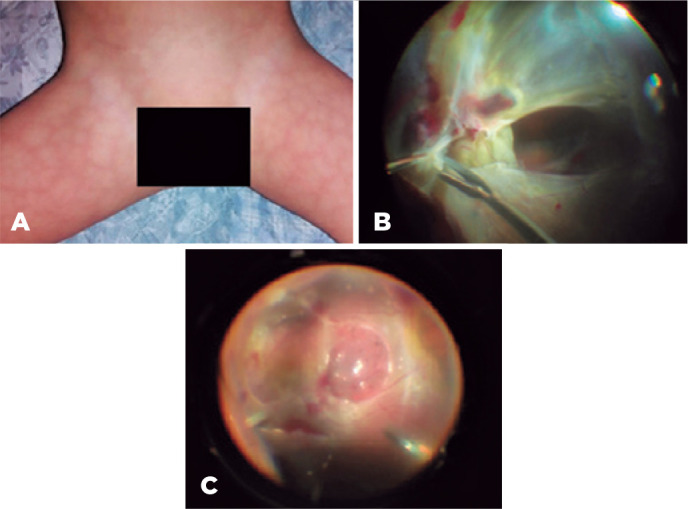
A) Bilateral and symmetrical netted skin lesions on the trunk and extremities. B and C) Intra-operative vitrectomy via pars plana with removal of macular traction. The optic disk, the posterior pole, and the middle periphery are difficult to visualize because of the presence of

A 2-year-old boy had a sudden visual loss in the OD since the second day of life. He lacked a history of surgeries, traumas, use of medications, or intercurrent disorders during pregnancy; he had had a term delivery. We found bilateral and symmetrical netted skin lesions on his trunk and extremities and shortening of the right lower limb. His visual acuity in the OD was followed up the examiner and did not in the OS, we found no biomicroscopic or IOP disturbances in either eye. On the fundoscopy, we found fibrovascular tissue with retinal tractions involving the maculae in both eyes (and with aspects of chronicity in the OS). We scheduled the patient for a pars plana vitrectomy in the OD, with retinal re-attachment and implantation of silicone oil (Figure 2).

## CASE 3

**Figure 3:**
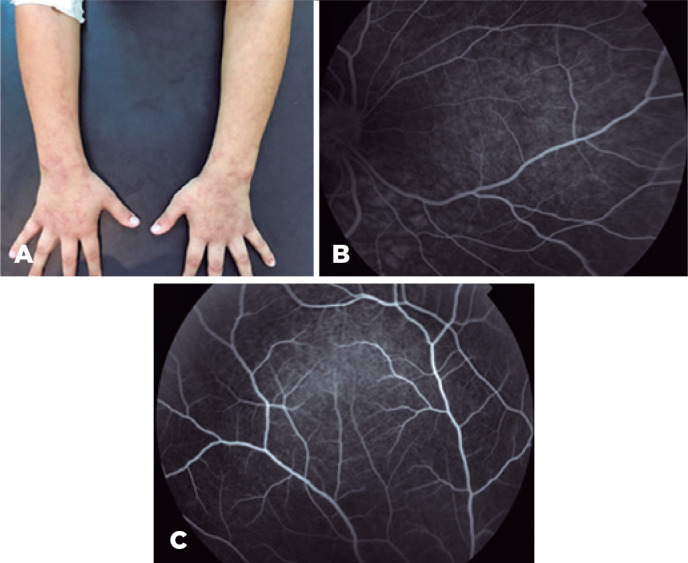
A) Livedo reticularis in limbs. B) Fluorescein angiography showing fibrovascular tissue and retinal traction. normal retinal perfusion, with absence of peripheral retinal ischemia.

An 8-year-old child with CMTC presented reticular, symmetric, and bilateral skin lesions in the trunk and limbs, and had a cortical cataract in the OS diagnosed at 4 years. The patient lacked a history of intercurrent disorders during gestation and had a normal development. Fundoscopies of both eyes were normal. Fluorescein angiography did not show changes in peripheral perfusion in either eye (Figure 3). We scheduled the patient for a facectomy and capsulotomy in the OS with visual acuity; after the operation, the best-corrected visual acuities were 20/20 in the OD and 20/30 in the OS.

## DISCUSSION

CMTC is a rare cutaneous disease, without a defined etiology or clinical diagnosis. The suggested diagnostic criteria require the presence of all three major criteria (congenital reticulated erythema, absence of venectasia, and non-responsivity of reticulated erythema to local warming) and at least two minor criteria (fading of erythema within two years, telangiectasia, port-wine stain outside of the affected area, cutaneous ulceration, or atrophy within affected areas)^(4)^. CMTC is associated with extracutaneous changes in more than 50% of cases (18.8%-89%)^(3)^, with unilateral body asymmetry being the most common extracutaneous manifestation^(4)^ characterized by hypertrophy or hypoplasia of limbs, trunk, or face^(6)^.

Ophthalmologic changes are rare and include congenital unilateral glaucoma (the most common ocular involvement), either associated or not to nevus flammeus and homolateral port wine stain^(3,5)^. The etiology of glaucoma is uncertain and may be related to abnormalities of the camerular sinus (similar to the case in primary congenital glaucoma), to episcleral vein dilation similar to that occurring in Sturge-Weber syndrome, or to neovascular glaucoma secondary to retinal detachment. The treatment is variable and involves eye drops, trabeculotomy, and trabeculectomy with drainage implants^(5)^.

The main CMTC-related retinal changes include exudative vitreoretinopathy with fibrovascular membrane and traction retinal detachment, vessel and optic disk dragging, congenital retinal detachment with neovascular glaucoma, peripheral avascular areas with associated vascular alterations, and neovascularization^(7,8)^. With the exception of neovascular glaucoma, these findings were present in the first two cases we presented. The recommended treatment for these manifestations is surgical intervention with pars plana vitrectomy^(2)^.

Other ophthalmologic alterations described in the literature are retinoblastoma, vascular changes secondary to ischemia associated with optic disk drusen, persistence of the hyaloid artery, small optical discs associated with cerebral atrophy, and cataract^(6,8)^, as we saw in the patient we described last.

Vascular changes, such as hemangiomas, nevus flammeus, telangiectasias, and phlebectasias, are commonly found in CMTC, in addition to cardiac and renal malformations, hypothyroidism, syndactyly, and bone dysplasias, among others^(3,4)^. Neurological abnormalities and developmental delays may be present, and when associated macrocephaly is found, the disease is named macrocephaly-cutis marmorata telangiectatica syndrome or macrocephaly -capillary malformations^(9)^.

The etiology of CMTC is unknown, its nonspecific histopathology and pathogenesis are probably multi factorial, most cases are sporadic, although rare fa milial complications can exist^(3,8)^. The differential diagnoses of CMTC are physiological cutis marmorata, Klippel-Trénaunay Syndrome, Sturge-Weber Syndrome, Adam-Oliver Syndromes, Bockenheimer’s Disease, Divry-Van Bogaert Syndrome, and collagenosis, as in neonatal lupus^(3,6,10)^.

The prognosis is good, with reduction of the cutaneous lesions usually within the first two years of life without treatment. However, for cases with extracutaneous involvement affecting the eyes (specifically the retina with peripheral ischemia), prophylaxes with laser photocoagulation should be considered^(1,4,8)^.

In conclusion, CMTC needs a fast diagnosis and a multimodal evaluation. Early evaluation by knowledgeable ophthalmologists is of great importance to avoid visual loss that can be irreversible.
